# Supplementary material for: Oxidative killing of encapsulated and nonencapsulated Streptococcus pneumoniae by lactoperoxidase-generated hypothiocyanite
Source: PLoS One. 2020 Jul 30;15(7):e0236389. doi: 10.1371/journal.pone.0236389 (PMC7392276; doi:10.1371/journal.pone.0236389)
Supplement: Attachment — Submitted filename: Gingerich Rada Plos One - Response to reviewers.docx [file pone.0236389.s001.docx]

Response to the critiques

We thank the editor for allowing a resubmission of this revised manuscript. We have made every effort to address the relevant comments. We hope that the manuscript is now acceptable for publication.

Responses to the editor's critiques:

We disagree that our findings are merely confirmative of previous findings. While several Streptococcus species have already been found to be sensitive to OSCN^-^, no published scientific literature is out there to show this for the clinically most important Streptococcus species, *Streptococcus pneumoniae*. I know this is surprising but we searched PubMed thoroughly and did not find any publication showing the effectiveness of the LPO-based system in any experimental setup against *S. pneumoniae*. We actually wrote the most up-to-date review in 2019 on the topic and listed all the 100+ publications studying several microbial species that have been studied for LPO-mediated killing, but *S. pneumoniae* is not there. We think our data is an important set of information completely suitable to your journal showing that the clinically most relevant Streptococcus species, *S. pneumoniae*, is sensitive to OCSN^-^ *in vitro*. This is also important because we have unpublished data showing that some Streptococcus species are not sensitive, so one cannot simply assume that all Streptococcus species are sensitive to OSCN^-^.

Regarding the criticism of not characterizing the *spxB*-deficient Spn mutant, there are actually data shown in the manuscript that SpxB is active under our experimental conditions in Fig. 7A as the *spxB*-deficient mutant had about 80% reduction in H_2_O_2_ production compared to the WT strain. The killing experiments were done under the same experimental conditions.

The editor is right, we forgot to add what medium was used in the growth assay. Bacteria were grown on BAP medium. This was added to the revised manuscript.

Response to the reviewer:

*Critique: How can the LPO system (LPO + H2O2 + SCN- = HOSCN) be a “novel, anti-pneumococcal therapy” when it is a principal component in the innate defense stratagem of the endocrine system, and there already exists an abundance of literature (100 + papers?) that evidence the effectiveness of the LPO system against streptococci, dating back more than 70 years.*

Response: As also detailed above in our answer to the editor, no one documented the effect of this system against *S. pneumoniae* (Pneumococcus) before. I agree that several other Streptococcal species were published but not *S. pneumoniae*. We cited them all in our review published in 2019 in the Journal of Microbiology (https://www.ncbi.nlm.nih.gov/pubmed/29858825). So, based on this, using/targeting the OSCN^-^based mechanism provides theoretically a novel strategy to kill pneumococcus, not other Streptococci. Several mechanisms of the immune system can be and have been targeted to improve bacterial clearance, so the same can be done with the LPO-based system. At the same time, we acknowledge that the results are restricted to an in vitro system.Therapeutic considerations might be overreaching; therefore, we removed this part of the discussion from the revised version.

The reviewer’s comment related to the endocrine system is unclear? We never said the LPO-based system is an endocrine system.

*Critique: the present paper is at best pedestrian. The claim that this is the first study of the efficacy of the LPO system vs. Streptococcus pneumoniae is false. Use the Google machine.*

Response: We used both the Google and PubMed “machines” and there is no publication to show that OSCN kills *Streptococcus pneumoniae*. The reviewer is more than welcome to refer to papers showing *Spn* killing by OSCN in the literature.

*Critique: Comically, the authors test a undefined "commercial product" by Profound Products that “stabilizes” HOSCN. According to the description the “1st Line” product, it also contains as “other” ingredients “hydrogen peroxide, poly aluminum chloride, lactoperoxidase and bentonite (note that no hydrogen peroxide or aluminum is consumed as these are converted by the enzyme in the manufacture of thiocynate (sp) ions).” We note that thiocyanate is not produced by these ingredients, and LPO is inactivated by hydrogen peroxide in the absence of thiocyanate. This reviewer has knowledge this product actually contains iodide, and HOSCN oxidizes iodide to yield iodine, the probable active ingredient.*

Response: We defined the product clearly in the methods section and only referred to it in the text as commercial product. We can provide any additional information, as much as needed. Of course, the 1^st^ Line product has LPO, H_2_O_2_, SCN^-^, polyaluminum chloride and bentonite in it –as they state in their description. Why would SCN^-^ be produced by these ingredients when it is provided as one of the substrates? This comment was unclear. We measured OSCN^-^ production with this kit and found it to be comparable to the levels obtained by the glucose/glucose oxidase system. SCN^-^ is provided in the kit, so LPO is unlikely to be inactivated by hydrogen peroxide in the absence of thiocyanate – as the reviewer stated it. We cannot comment on the undocumented knowledge the reviewer claims to have on this product. No data are shown or referred to.

Critique: l.128: The LPO system described effectively generates a bolus of HOSCN. It is not clear if the HOSCN was generated exogenously, then added to the wells.

Response: We might have different definitions for “bolus”. When we used the term “bolus”, the compound was not produced by enzymatic reaction but provided already synthesized in an unnaturally high concentration in one moment. HOSCN was generated in the wells, as described in the methods.

Critique: l.133: It is not at all clear what grown medium was used. Was the medium just HBSS or BAP? If HBSS, this stressor would not realistically reflect the infectious agent in vivo. HOSCN reacts instantly with BAP.

Response: HBSS was used in the killing assay as stated in the methods and bacteria were then inoculated on BAP for growth. This experiment uses an in vitro system with its obvious limitations and advantages as a model of the in vivo environment. Similar buffers were used in most of the published papers using such in vitro systems to study the antimicrobial effect of this LPO-based system. The information gathered is useful to document that another bacterial species, *S. pneumoniae* is also killed by the LPO/SCN^-^ system. How much is this true and reflected in vivo, remains to be clarified not only for *S. pneumoniae*, but for all the other pathogens published since –paradoxically- the relevance of this antimicrobial system has not yet been established in any mammalian organism including mice and humans. We added more information about the growth conditions of bacteria to the revised manuscript.

Critique: l.193: “… short-lived TNB cannot be purchased and was generated by reducing DTNB with the help of beta-mercaptoethanol”. First, TNB is not short-lived. We have a flask of it that was synthesized more than a decade ago and shows no sign of decomposition. Second, if TNB was synthesized from DTNB in situ using ME, there is a likelihood that unreactive ME remains, which would render the assay inaccurate.

Response: We read several publications that measured OSCN^-^ production by this simple method and all claimed that TNB is not stable over long period of time. We, therefore, decided to purchase DTNB and reduce it with the help of ME, as dozens of related manuscripts did it too.

Critique: l.237: “H2O2 is provided by the enzymatic reaction of glucose oxidase with glucose, not in a bolus-like fashion.” … the authors clearly do not understand enzyme kinetics. I calculate a half-life of about 18 seconds for this reaction under the conditions employed.

Response: We actually backed up our decision for using this method of H_2_O_2_ production with more meaningful, and for the studied topic, more useful hydrogen peroxide measurements. Adding 1 mM bolus H_2_O_2_ to the system -as is done by several investigators in the field- has nothing to do with the nature of H_2_O_2_ production by their physiological source, bronchial epithelial cells. We measured and optimized H_2_O_2_ production of the glucose/glucose oxidase system so that it mimics the slow but relatively steady nature and amplitude of H_2_O_2_ production by airway cells. While it is true that –of course- the rate of H_2_O_2_ production slowed over time, it was still detectable at 60 minutes. Despite its deceleration over time, it is still a better approach to model H_2_O_2_ generation by airway cells than pouring a bucket load of physiologically irrelevant concentration of H_2_O_2_ into our system.

*Critique*: l.249: Yes, catalase, the most efficient enzyme known to man, removes one of the essential components of the LPO system.

Response: While the effect of catalase is obvious, one cannot simply assume that it will also work in our experimental system and has to show results. Removing a critical component of a system is an essential approach to show how the system works and this approach should rather be appreciated and encouraged by the reviewer to show the H_2_O_2_-dependence of the studied mechanism.

Critique: l. 278: Figure 3B exhibits a profound ignorance of the LPO system. Under the conditions described in the experimental section, only [SCN-] > 50 uM will produce HOSCN under their reaction conditions. Also, the assay does not test for regrowth (the conditions are likely inhibitory, not cytocital).

Response: I do not see any ignorance of the LPO system here and in fact the reviewer’s calculations clearly confirm that OSCN^-^ is responsible for the inhibitory effect of microbial growth observed in Fig 3B as the lowest SCN^-^ concentration used is 40 uM. The second observation is not new, at all, as we clearly stated in the manuscript that the growth assay used in figure 3B measures inhibition of bacterial growth and not direct microbicidal activity. We had the CFU assay for that.

Critique: l.337: Been shown before.

Response: No, it has not. Please cite any report from the medical literature and we will immediately retract our submission.

Critique: l.401: HOSCN only attacks sulfhydryl groups.

Response: That’s what we said too, so we are unclear about the point of this critique. In line 401 we referred to the long list of microbes OSCN^-^ has been described to target, if that was confusing.

Critique: l.407: So is bleach.

Response: Natural products of the immune system have been and are being used as part of therapies for a long list of diseases.

Critique: .411: I am aware of at least two patents for the use of the LPO system in the treatment of lung infections.

Response: So are we. We cited them in our last year’s review in Journal of Microbiology. None of them, however, claims to be targeting *S. pneumoniae*. We refer to its novelty to be used against Pneumococcus, not other bacteria.

Critique: I would like to know if the authors have received any financial support from Profound Products.

Response: No, we have not. We would have acknowledged it in the manuscript if we had according to the policies of *PLoS One* and any other journal. We used this product because this was the only, purchasable product we could find. We are glad to test any other, commercially available OSCN-producing product in the future.
