# Supplementary material for: Oxidative killing of encapsulated and nonencapsulated Streptococcus pneumoniae by lactoperoxidase-generated hypothiocyanite
Source: PLoS One. 2020 Jul 30;15(7):e0236389. doi: 10.1371/journal.pone.0236389 (PMC7392276; doi:10.1371/journal.pone.0236389)
Supplement: Attachment — Submitted filename: Gingerich Rada Plos One - Response to reviewers.docx [file pone.0236389.s002.docx]

Response to the critiques

We thank the editor for allowing a resubmission and re-review of our revised manuscript. We also thank the reviewers for their constructive criticisms of our manuscript that have significantly improved it. We made every effort to address all the comments. We hope that the manuscript is now acceptable for publication.

- Response to the editor:

According to the editor’s request, we made sure that the manuscript meets PLOS ONE’s style requirements, including those for file naming. The single data set that was referred to as “data not shown” in the original, not the revised, version of the manuscript has been removed. No need, therefore, for supplementary data deposition. We are also attaching and uploading the minimal data set as a Supporting Information file requested by your journal.

- Responses to comments of reviewer 2:

*Critique: Line 409. Change ‘Spxb’ to ‘SpxB’*

*Response*: Thanks for pointing this out, we made the change in the revised text.

*Critique: Discussion. It might be relevant to add a paragraph on limitations/technical challenges that will need to be addressed before the product can be used in vivo – these may well be future experiments that the authors would like to conduct. There is clear antibacterial efficacy in vitro but translation to in vivo efficacy will be a challenge, and something that most readers will contemplate.*

*Response*: We thank the reviewer for bringing this important issue up. According to this, a separate paragraph has been added to the end of the discussion to mention all the limitations of our current study using a cell-free, in vitro experimental system.

- Responses to the comments of reviewer 3:

*Critique: For all figures, it is unclear if the reported data represents technical or biological replicates.*

*Response*: We thank the reviewer for this request. We added this information to all the figure legends in the revised manuscript.

*Critique: For all bacteria killing assays assessed by CFU, the methods section states that "A time zero condition was also counted to make sure that bacterial death was due to OSCN- and not related to an unknown variable". It is agreed that is an important control, but these time zero CFU counts do not appear to be reported in any of the related figures (1A, 5, 6, 7C). Please include this data.*

*Response*: We thank the reviewer for this comment. While we agree that this data serve as important controls, we consider them quality controls of the assay, therefore, decided not to report them individually at each figure but to expand the related sentence in the description of the CFU killing method to this:

“A time 0 condition was also counted to make sure that bacterial death was due to OSCN- and not related to an unknown variable, and that no significant changes in bacterial numbers were observed in samples containing only bacteria during the duration of the experiments.”

Critique: *In some instances, data derived from the bacteriostatic assay (where growth inhibition is measured by OD in a microtiter plate assay) is incorrectly referred to or discussed as demonstrating "killing", when in fact this assay is measuring growth inhibition. (ex: Figures 2 and 3 legend titles, results line 289).*

Response: We tried to be consistent with the related nomenclature of “killing” vs “growth inhibition” throughout the manuscript but obviously missed these few occasions. We thank the reviewer for identifying them. The terms “killing” or “killed” were changed in both instances to “growth inhibition”.

*Critique: For all figures reporting CFU data, converting the Y axis to log scale would improve resolution of the actual CFU data points that cluster around the "0" Y axis point (ex: when bacteria are incubated in the presence of OSCN-).*

*Response*: We had long discussions in the group whether the linear or log scale results should be presented in the figures with CFU data. We had decided to present the results on linear scale, instead of log scale, because we think it better shows the impressive antibacterial effect of OSCN- against Spn.

*Critique:* *The data presented in Figure 5 suggest that in most cases, the catalase control worked better in the capsule mutants compared to their parental strains; is there a reason this might be the case?*

*Response*: We thank the reviewer for this interesting observation. Indeed, catalase worked better in case of TIGR4 and D39 in the absence of the capsule while this difference was less pronounced in case of EF3030 (Fig. 5). While we do not have any proven explanation for this at this point, we have added a few sentences about this to the discussion pointing to potential explanations.

*Critique: For the experiment in Figure 7 (comparing wild0type and pyruvate oxidase mutant killing by OSCN-), why was the "1st line" product used rather than the in vitro assay used in Figure 1? The fact that the mutant was more susceptible to OSCN- is very interesting. Was this phenotype genetically complemented? Figure 7C is somewhat confusing, why not present the Y axis data as CFU counts?*

*Response:* In figure 6 and 7 we did not only want to test the effect of the respective bacterial mutants but also aimed at delivering the first results to show that “1^st^ line” is not only bacteriostatic but also directly kills Spn. This was actually mistakenly stated in the figure 6 legend and now it has been corrected to “1^st^ line”. A sentence was added about the direct Spn-killing effect of 1^st^ line to the figure 6 section of the results.

No, the SpxB-deficient mutant was not complemented.

We would like to keep the “susceptibility to OSCN’” term as introduced in figure 7 as we think it better explains the antimicrobial action of OSCN- than showing the CFU results. We are aware of it that it might get somewhat confusing, therefore we added an extra explanation to the corresponding part of the results section.

*Critique:* *Results line 339: the term "allolysis" (instead of autolysis) is more appropriate when discussing in this context.*

*Response:* We thank the reviewer for the comment. However, we feel in the overall context of the manuscript autolysis is the response we tested and chose not to change the wording. Classically, autolysis is associated with lytA expression and lysis of self, while allolysis is more a term of fratricide (lysis of nearby cells). Allolysis is also associated with the competence system and includes up to seven genes. In our study, we used a specific lytA gene knock out strain to look at the effects of autolysis. We did not look at the impact of differences in competence or fratricide in the in vitro model of Spn killing, so allolysis is not an appropriate term to be used based on our experimental approach.
